# Behavioral and Environmental Factors of Carbon Monoxide Poisoning and Polycythemia due to Waterpipe Smoking: An Artificial Intelligence-Assisted Systematic Review of Case Reports

**DOI:** 10.31662/jmaj.2025-0208

**Published:** 2026-02-20

**Authors:** Isao Muraki

**Affiliations:** 1 Department of Public Health Medicine, Institute of Medicine, and Health Services Research and Development Centre, University of Tsukuba, Tsukuba-shi, Japan; 2 Public Health, Osaka University Graduate School of Medicine, Osaka, Japan

**Keywords:** waterpipe smoking, carbon monoxide poisoning, polycythemia, systematic review, case reports

## Abstract

**Background:**

We aimed to explore behavioral and environmental factors associated with carbon monoxide (CO) poisoning from waterpipe smoking.

**Methods:**

We searched the MEDLINE, Web of Science, PubMed, CINAHL, Ichushi-Web, and CiNii databases for case reports or case series on acute CO poisoning and polycythemia related to waterpipe smoking, up to February 2025. Article selection and data extraction were performed with the assistance of generative artificial intelligence (AI) using predefined criteria. All processes performed by AI were verified by the author through careful article review.

**Results:**

A total of 68 cases of acute CO poisoning and 13 cases of polycythemia were identified from 37 case reports and series. Most cases involved individuals aged 20‒29 years. The most common symptoms of acute CO poisoning following waterpipe smoking were syncope, headache, dizziness, and nausea/vomiting, in that order. Symptoms typically occurred shortly after exposure, followed by during exposure, and delayed after exposure. Acute CO poisoning was observed after as little as one hour of waterpipe smoking, in outdoor settings, and from secondhand waterpipe smoking exposure.

**Conclusions:**

Both active and passive waterpipe smoking can cause acute CO poisoning, depending on the intensity, duration, and environment of exposure. Symptoms may arise not only during or immediately after smoking but also with a delayed onset. The implementation of environmental regulations for closed establishments is necessary to prevent mass CO poisoning and protect workers from it. Further research is needed to better understand the behavioral and environmental factors contributing to CO poisoning from waterpipe smoking.

## Introduction

**Figure 1.**
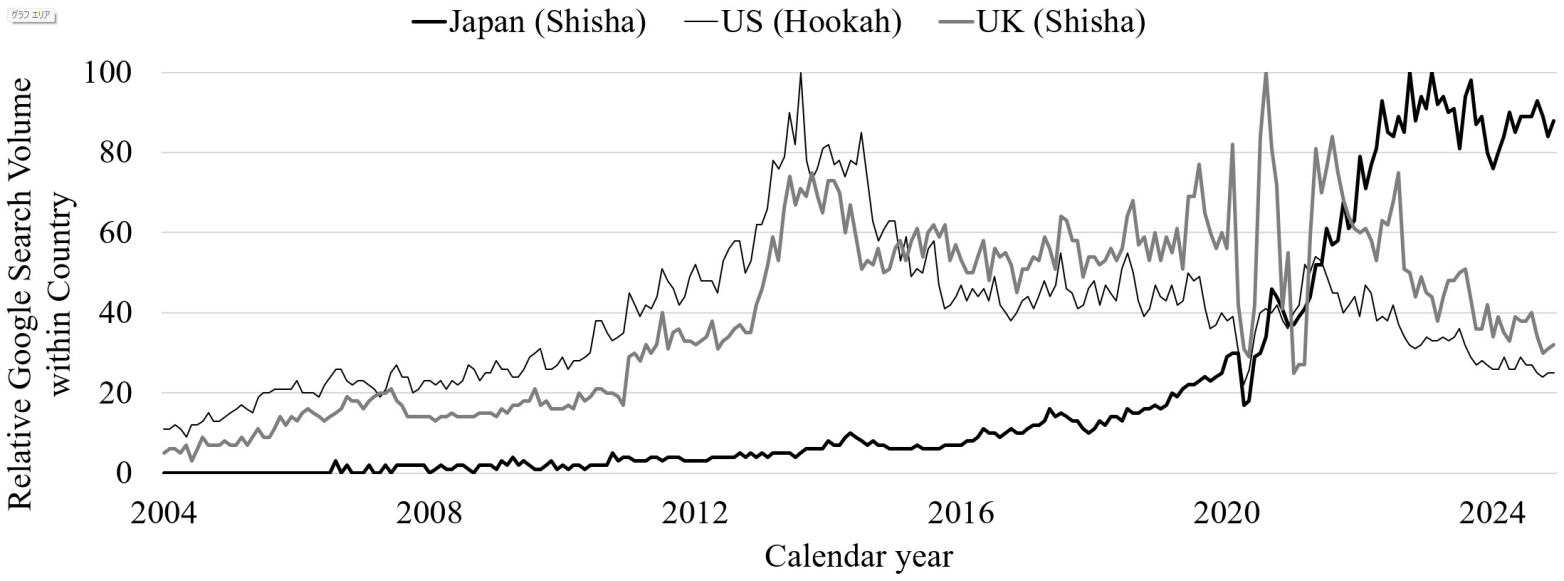
Trend of monthly relative Google search volume for shisha (Japan and U.K.), and hookah (U.S.) from Google Trends data from 2004 to 2024. The thick black line indicates the Japanese trend, the thin black line indicates the U.S. trend, and the thick gray line indicates the U.K. trend. The value represents the monthly search volume divided by the maximum monthly search volume in each country between January 2004 and December 2024. The values are not comparable between countries. The maximum monthly search volume (100) is in February 2023 for Japan, August 2013 for the U.S., and August 2020 for the U.K.

Since the early 2000s, interest in waterpipe smoking has risen in Western countries ^(1)^, particularly among adolescents and young adults in the U.S. ^(2)^. Over a decade later, interest in waterpipe smoking also surged in Japan (Figure 1). Waterpipe smoking has been associated with an increased risk of tobacco smoking ^(2)^ and marijuana use ^(3)^, as well as waterpipe-related poisoning incidents ^(4)^. Therefore, waterpipe smoking has been a global health concern.

Acute carbon monoxide (CO) poisoning following waterpipe smoking is a unique waterpipe-related health hazard. This poisoning can cause various symptoms, including syncope and headaches in the acute phase, as well as delayed neurological effects occurring between two and 60 days after exposure. Waterpipe-related poisoning incidents have risen in parallel with the growing popularity of waterpipe smoking among adolescents and youths in the U.S ^(1), (4)^. In line with the rising interest in Japan, the first reported case of acute CO poisoning from waterpipe smoking occurred in 2017 ^(5)^, and the number of such cases has been increasing since then. Between January 2018 and June 2023 (66 months), 64 patients in the three districts of the Tokyo Metropolitan Area (Meguro, Setagaya, and Shibuya) were transported to hospitals by ambulance due to acute CO poisoning ^(6)^. Two previous epidemiological studies examined the clinical manifestations of acute CO poisoning from waterpipe smoking compared to other causes ^(7), (8)^. However, no epidemiological study has examined the association between behavioral and environmental factors of waterpipe smoking and CO poisoning.

Therefore, this study aimed to systematically review case reports on acute CO poisoning and polycythemia (chronic CO poisoning) linked to waterpipe smoking to identify potential behavioral and environmental risk factors, such as active or passive waterpipe smoking, smoking duration, and the closedness of the smoking location.

## Materials and Methods

### Overview of workflow

We systematically reviewed all case reports of CO poisoning or polycythemia associated with waterpipe smoking. First, the author conducted a comprehensive search. Then, the selection and data extraction processes were conducted independently by a human (the author) and generative artificial intelligence (AI). We developed and refined the prompts for selection and data extraction using sample data through trial and error. The same prompt was repeatedly used for each task. The author verified all processes performed by the generative AI. Data extraction was performed by the generative AI first, and the author verified and corrected the extracted data by carefully reviewing the manuscript. Finally, the author analyzed the data and prepared the manuscript. All tables and figures were created by the author.

### Study approval and registration

As this study was a secondary data analysis of published articles, ethics committee review was not required. Although we prepared the review protocol before searching the database, this study was not registered in the PROSPERO database because the requirements for the number of review team members (two or more members were required) were not met.

#### Eligibility criteria, information sources, and search strategy

A comprehensive search was conducted across four international databases (MEDLINE, Web of Science, PubMed, and CINAHL) and two Japanese databases (Ichushi-Web and CiNii) without language restrictions, covering articles published up to February 2025. The search terms used were: (‘Waterpipe’ OR ‘Water pipe’ OR ‘Shisha’ OR ‘Hookah’ OR ‘Narghile’) AND (‘Carbon monoxide poisoning’ OR ‘Carbon monoxide intoxication’ OR ‘Polycythemia’). In MEDLINE and PubMed searches, Medical Subject Headings (MeSH) terms such as ‘Smoking Water Pipes,’ ‘Carbon Monoxide Poisoning,’ and ‘Polycythemia’ were also included in addition to the search terms mentioned above. Conference abstracts were excluded due to their limited information and availability.

### Selection process and data extraction

The selection process involved both human and AI-assisted screening. The inclusion criteria were as follows: 1) a description of a waterpipe (hookah, shisha, or narghile) or its symptoms in the title or abstract; 2) a description of CO poisoning or polycythemia in the title or abstract; and 3) the article type was case reports or case series. First, the author (IM) screened all titles and abstracts to identify candidate articles on CO poisoning or polycythemia related to waterpipe smoking. Then, similar to the human process, the author used generative AI GPT o3-mini (ChatGPT Pro, OpenAI, Inc., San Francisco, California) on March 10, 2025, to screen titles and abstracts using the first predefined prompt according to the inclusion criteria (Supplemental Material 1). When the article type was a case report or case series, articles selected by either the author or AI were included as candidates to minimize erroneous exclusion.

Next, before the data extraction by AI, the author reviewed the full manuscripts screened by title and abstract to confirm whether they contained case information. For non-English and non-Japanese articles, the author used the online DeepL translator (DeepL SE, Cologne, Germany) to translate the text into English, assuring the accuracy of interpretation by ChatGPT for translation. Then, GPT o3-mini-high (ChatGPT Pro, Open AI, Inc., San Francisco, California) was used on March 11, 2025, to extract case details [authors, publication, publication year, language, country, patient ID, age, sex, case types, shisha use, active user, co-user number, place, room size, time using shisha, using length (hrs), nicotine-free, shisha café/bar worker, alcohol use, habitual shisha use frequency, symptoms, clinical information, ambulance transport, Glasgo Coma Scale (GCS), Japan Coma Scale (JCS), neurological exam, physical exam, brain computed tomography (CT), brain magnetic resonance imaging (MRI), vital signs, measured time after smoking, peripheral carboxyhemoglobin saturation (SpCO) at triage, SpCO after atrial blood gas (ABG), carboxyhemoglobin (COHb) at triage, COHb after ABG, pH, pCO_2_, pO_2_, HCO_3_] from the full manuscript using the second predefined prompt (Supplemental Material 2). When waterpipe use details (exposure duration and frequency) or the diagnosis of acute CO poisoning or polycythemia were not described, the article was excluded, as it did not meet the criteria for a case report or case series. When mismatches existed between the AI- and author-selected articles, the author carefully reviewed the mismatched articles again and made the final decision.

Data extraction was performed as part of the selection process using ChatGPT. The author verified the extracted data one by one through a manual review of all original manuscripts. The quality of the included articles was assessed based on the richness of information across five aspects: demographics (age and sex); waterpipe use (habitual use, use before onset, duration, location, and co-users); symptoms (onset and symptom description); clinical findings (mode of arrival, physical examinations related to acute CO poisoning [GCS, blood pressure, pulse, respiratory rate, and neurological examinations], and diagnostic assessments [SpCO or COHb levels and blood hemoglobin levels]); and treatment (type of treatment and post-treatment measurements of COHb or hemoglobin levels), as shown in Supplementary Table 1. We counted the number of available items for each category. To avoid excessive weighting of physical examinations, we first scored the physical examination items before assigning the overall clinical findings score (zero points for 0-1 item, one point for 2-3 items, and two points for 4-5 items). For case series, the average score across all cases was used to assess overall quality. The scoring criteria were as follows: demographics and symptoms (0 = low, 1 = medium, 2 = high); waterpipe use (1 = low, 2 = medium, 3-5 = high); clinical findings (1 = low, 2-3 = medium, 4-5 = high); and treatment (0 = low, 1 = medium, 2 = high).

### Data synthesis and analysis

Extracted data were summarized by calculating the number of cases according to age, sex, waterpipe use (frequency, duration, and location), symptoms, and timing of symptom onset. Missing data were treated as missing. Additionally, the mean levels of COHb and hemoglobin were calculated from the valid data. The duration of waterpipe use before symptom onset was categorized into five groups: during exposure; shortly after exposure; delayed after exposure; no apparent symptoms; and missing. The “delayed after exposure” group included cases that occurred after leaving the exposure location, or one hour or longer after use. For the duration of waterpipe use, the response “several hours” was treated as “2 to <3 hours”.

## Results

**Figure 2.**
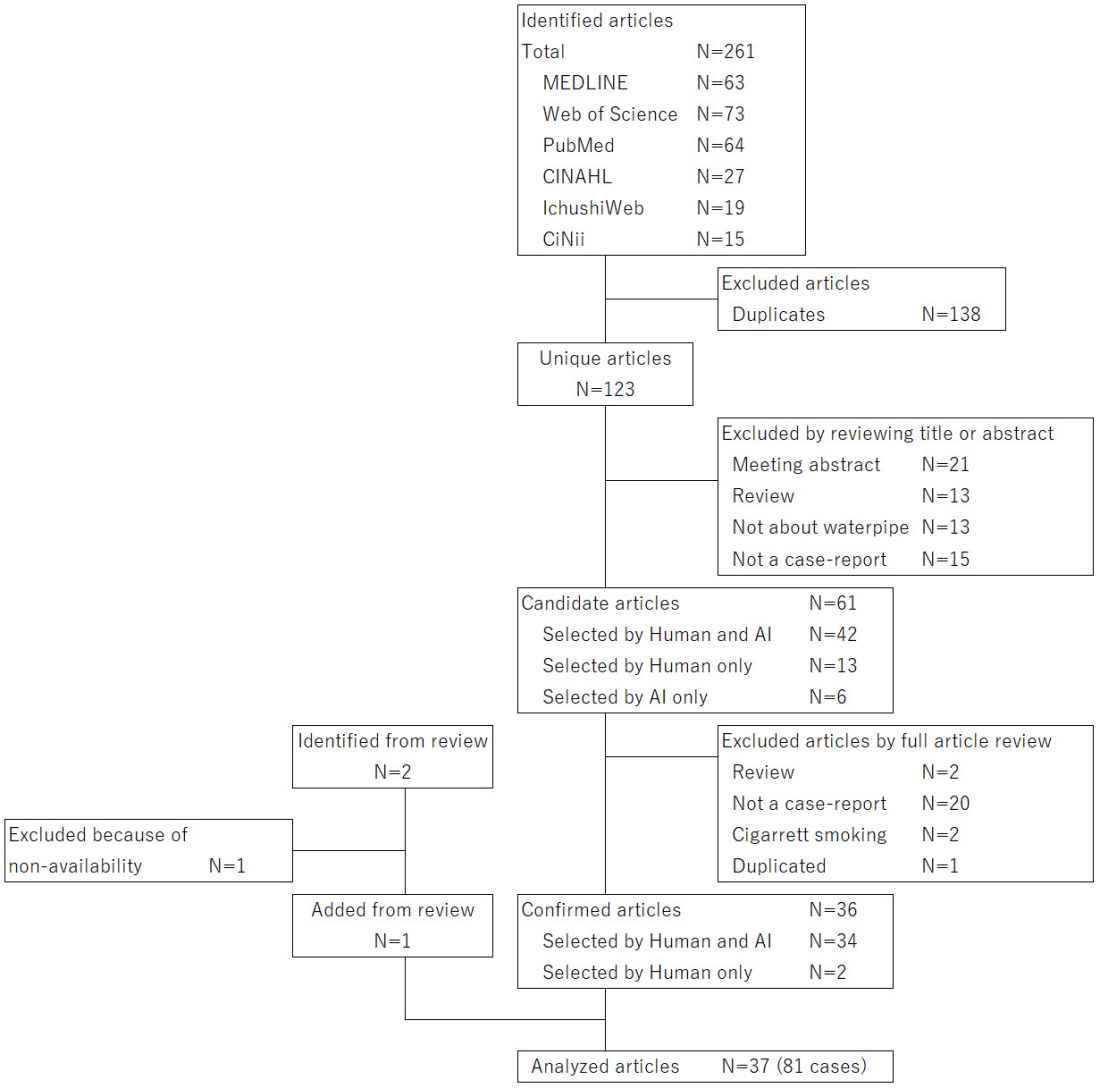
Flowchart of included article selection.

This systematic review identified a total of 81 cases of acute CO poisoning or polycythemia caused by waterpipe smoking, documented in 37 case reports and series (Figure 2) ^(5), (9), (10), (11), (12), (13), (14), (15), (16), (17), (18), (19), (20), (21), (22), (23), (24), (25), (26), (27), (28), (29), (30), (31), (32), (33), (34), (35), (36), (37), (38), (39), (40), (41), (42), (43), (44)^. One additional article identified from a review was unavailable, as it was accessible only on a French-language website ^(45)^. A detailed list of cases, categorized by diagnosis and exposure (active or passive waterpipe smoking), is provided in Supplemental Table 1. The quality of case series and reports included was shown in Table 1. More than half of the case series and reports contained limited key information on symptoms due to missing data on symptom onset. Nearly three-fourths of the clinical findings were poorly reported. The case series appeared to be of relatively low quality. There were no apparent temporal or areal differences in quality.

**Table 1.**
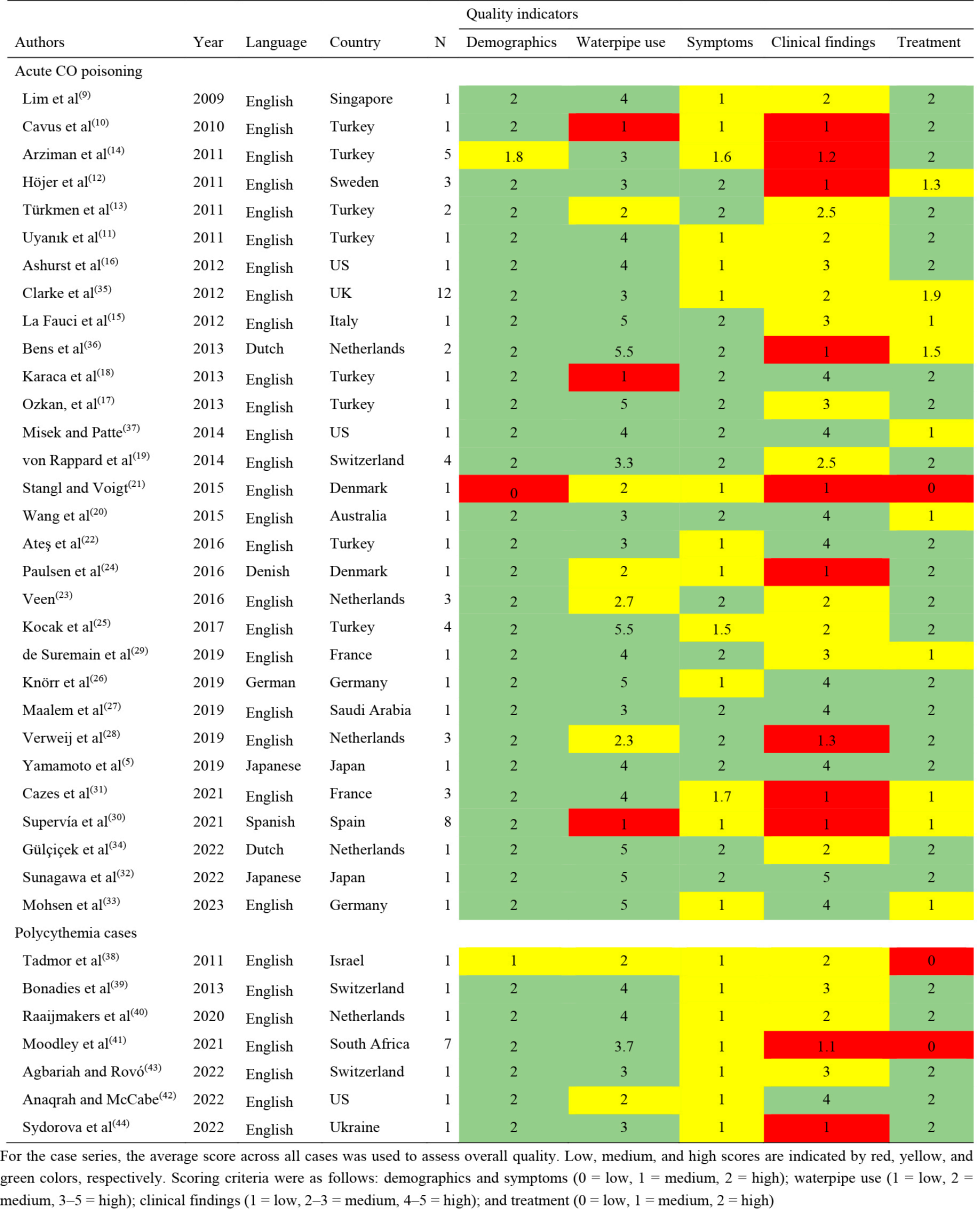
Quality Assessment of Included Cases Series and Reports with Acute Carbon Monoxide Poisoning and Polycythemia.

Among the 81 identified cases, 68 were of acute CO poisoning ^(5), (9), (10), (11), (12), (13), (14), (15), (16), (17), (18), (19), (20), (21), (22), (23), (24), (25), (26), (27), (28), (29), (30), (31), (32), (33), (34), (35), (36), (37)^ and 13 of polycythemia ^(38), (39), (40), (41), (42), (43), (44)^ (Table 2). Case reports of acute CO poisoning appeared in 2009 ^(9)^ and have increased since then. Among the 68 acute CO poisoning cases, 64 resulted from active waterpipe smoking ^(5), (9), (10), (11), (12), (13), (14), (15), (16), (17), (18), (19), (20), (21), (22), (23), (24), (25), (26), (27), (28), (29), (30), (31), (32), (33), (34), (35), (36)^, while four were caused by secondhand waterpipe smoking exposure ^(25), (28), (31), (37)^. Two of the four passively exposed cases involved workers in establishments that provided waterpipe smoking services ^(25), (37)^. Acute CO poisoning due to waterpipe smoking was most commonly reported in European countries (66.2%), followed by Middle Eastern countries (25.0%). In contrast, polycythemia associated with waterpipe smoking was primarily reported in African countries (53.8%), followed by European countries (30.8%). Most cases for both acute CO poisoning and polycythemia occurred in individuals aged 20‒29 years. However, the second most affected age group differed across these conditions: 10‒19 years for acute CO poisoning and 30‒39 years for polycythemia. The male-to-female ratio was lower in patients with acute CO poisoning than in those with polycythemia. The most common symptoms of acute CO poisoning following waterpipe smoking included syncope (52.9%), headache (50.0%), dizziness (44.1%), nausea or vomiting (38.2%), and impaired consciousness (13.2%). The most common symptoms of waterpipe-related polycythemia were impaired consciousness (23.1%), deep vein thrombosis (15.4%), and facial erythema (15.4%). Among the 33 cases for which symptom onset timing was reported, symptoms occurred shortly after exposure in 16 cases (48.5%), during exposure in nine cases (27.3%), and with a delay after exposure in eight cases (24.2%). Injuries were reported in five cases: one occurred during exposure ^(31)^, one shortly after exposure ^(28)^, two were delayed ^(25), (29)^, and one had unclear timing ^(9)^. The mean COHb levels upon hospital arrival were similar between patients with acute CO poisoning (22.2%) and those with polycythemia (24.6%). Hemoglobin levels were rarely reported in patients with acute CO poisoning (three out of 68 cases) but were reported in all cases of polycythemia (13 out of 13 cases).

**Table 2.** Included Cases with Acute CO Poisoning and Polycythemia.

	Acute CO poisoning	Polycythemia
Number of cases	68	13
Country, n (%)		
North America	2 (2.9)	1 (7.7)
Europe	45 (66.2)	4 (30.8)
Middle East	17 (25.0)	1 (7.7)
Africa	0 (0.0)	7 (53.8)
Oceania	1 (1.5)	0 (0.0)
Asia	3 (4.4)	0 (0.0)
Age, n (%)		
10-19 years	22 (32.4)	0 (0.0)
20-29 years	33 (48.5)	6 (46.2)
30-39 years	8 (11.8)	3 (23.1)
40-49 years	3 (4.4)	1 (7.7)
50-59 years	0 (0.0)	1 (7.7)
Missing	2 (2.9)	1 (7.7)
Sex, N (%)		
Men	40 (58.8)	12 (92.3)
Women	27 (39.7)	1 (7.7)
Missing	1 (1.5)	0 (0.0)
Symptoms, N (%)		
Syncope	36 (52.9)	0 (0.0)
Headache	34 (50.0)	1 (7.7)
Dizziness	30 (44.1)	1 (7.7)
Nausea or vomiting	26 (38.2)	0 (0.0)
Weakness or collapse	10 (14.7)	1 (7.7)
Impaired consciousness	9 (13.2)	3 (23.1)
Injuries	5 (7.4)	0 (0.0)
Shortness of breath	4 (5.9)	0 (0.0)
Vertigo	3 (4.4)	0 (0.0)
Chest pain	3 (4.4)	0 (0.0)
Seizure	3 (4.4)	0 (0.0)
PE or DVT	0 (0.0)	2 (15.4)
Facial plethora	0 (0.0)	2 (15.4)
Others	17 (25.0)	2 (15.4)
None	2 (2.9)	2 (15.4)
Missing	0 (0.0)	0 (0.0)
Onset of symptoms, N (%)		
During exposure	9 (13.2)	0 (0.0)
Shortly after exposure	16 (23.5)	0 (0.0)
Delayed after exposure	8 (11.8)	0 (0.0)
No apparent symptoms	2 (2.9)	2 (15.4)
Missing	33 (48.5)	11 (84.6)
COHb levels		
Number of valid cases	65	6
Mean (SD)	22.2 (8.4)	24.6 (10.9)
Missing, N (%)	3 (4.4)	7 (53.8)
Hb levels		
Number of valid cases	3	13
Mean (SD)	15.5 (1.6)	20.5 (2.0)
Missing, N (%)	65 (95.6)	0 (0.0)

CO: carbon monoxide; COHb: carboxyhemoglobin; DVT: deep vein thrombosis; Hb: hemoglobin; PE: pulmonary embolism; SD: standard deviation.

Among the 68 acute CO poisoning cases following waterpipe smoking, 41 were single cases of active smoking ^(5), (9), (10), (11), (12), (13), (14), (15), (16), (17), (18), (19), (20), (21), (22), (23), (24), (25), (26), (27), (28), (29), (30), (31), (32), (33), (34)^, 23 were mass poisonings after active smoking at the same occasion ^(14), (19), (30), (35), (36)^, and four cases resulted from secondhand waterpipe smoking exposure ^(25), (28), (31), (37)^. The behavioral and environmental factors related to waterpipe smoking among individuals who smoke actively are shown in Table 3. In most case reports (58 out of 64 cases), habitual waterpipe smoking frequency before the incident was not specified. The duration and location of waterpipe smoking before CO poisoning were documented in 45-60% of cases. Among the 29 cases with duration information, six (20.7%) involved CO poisoning after only a brief period (<1 hour) of smoking ^(12), (14), (24)^. Among the 39 cases with information on smoking location, CO poisoning in 32 cases occurred indoors ^(5), (9), (11), (13), (14), (17), (19), (21), (22), (23), (25), (26), (29), (31), (32), (33), (34)^, while that in seven cases occurred outdoors ^(12), (15), (16), (27), (36)^. Mass poisoning at the same event was primarily reported in closed environments, such as basements and waterpipe cafés. The mean COHb levels were lower in mass poisoning incidents (16.2%) than in single-case incidents (25.5%). Regarding secondhand waterpipe smoking, two of the four affected individuals were employees in waterpipe cafés, bars, or lounges ^(25), (37)^. Additionally, one case of CO poisoning was reported when an individual entered a room where another person had smoked a waterpipe before she entered ^(31)^. Only one case report mentioned alcohol intake during waterpipe use ^(16)^, while four case reports did not mention any alcohol intake ^(15), (26), (32), (34)^. The other case series and reports did not provide information on alcohol consumption. Additionally, co-use of other substances, such as cannabis and marijuana, was not reported in any case series or reports.

**Table 3.** Exposure Characteristics of Active Waterpipe Smoking before Acute Carbon Monoxide Poisoning.

	n (%)
Number of cases	64
Intensity of waterpipe use before symptoms occurrence	
First time	1 (1.6)
3 consecutive days	1 (1.6)
7 consecutive days	1 (1.6)
Yes, but no details available	1 (1.6)
Missing	60 (93.8)
Duration	
<1 hour	6 (9.4)
1 to <2 hours	8 (12.5)
2 to <3 hours	4 (6.3)
3 hours or longer	11 (17.2)
Missing	35 (54.7)
Location	
Indoor	32 (50.0)
Private room	19 (29.7)
Restaurants, cafés, and bars	13 (20.3)
Outdoor	7 (10.9)
Missing	25 (39.1)
Habitual waterpipe use frequency	
Daily	1 (1.6)
3-4 times/week	2 (3.1)
2 times/month	1 (1.6)
Occasional	4 (6.3)
Yes, but no details available	1 (1.6)
No	1 (1.6)
Missing	55 (85.9)

Most patients with polycythemia (12 out of 13 cases) smoked waterpipes daily ^(38), (39), (40), (41), (42), (43)^. The least frequency of waterpipe smoking in polycythemia cases was every other day ^(44)^.

## Discussion

To the best of our knowledge, this systematic review of case reports is the first study to summarize the timing of symptom onset following waterpipe smoking and the behavioral and environmental factors associated with waterpipe-related CO poisoning.

Reports of acute CO poisoning due to waterpipe smoking have increased in European countries since 2007 ^(45)^. During the late 2000s, interest in waterpipe smoking grew moderately in Western countries ^(1)^, and a concurrent rise in waterpipe-related poisonings was noted in the U.S. ^(4)^. The spreading of waterpipe smoking is consistent with the increase in the number of case reports of acute CO poisoning related to waterpipe smoking observed in this systematic review. Acute CO poisoning has predominantly been reported in adolescents and young adults, which aligns with the known prevalence of waterpipe smoking in these age groups ^(2)^. As reported previously ^(7), (8), (46)^ and in the current findings, the four most common symptoms of acute CO poisoning following waterpipe smoking are syncope, headache, dizziness, and nausea/vomiting. Notably, compared with CO poisoning from other causes, syncope appears more frequently in waterpipe-related cases ^(7), (8)^.

Regarding the timing of symptom onset, one-fourth of acute CO poisoning cases exhibited delayed symptoms after waterpipe smoking ^(12), (19), (25), (29), (32)^. Notably, these individuals were more likely to experience injuries compared to those who had symptoms during or shortly after exposure. A delay in symptom onset may increase the risk of falls or other injuries, such as falling downstairs or onto hard surfaces. To mitigate this risk, individuals who engage in waterpipe smoking should be monitored in fresh air for a period after smoking.

Approximately half of the reviewed case reports described the duration of waterpipe smoking before symptom onset ^(5), (9), (11), (12), (13), (14), (15), (17), (19), (20), (22), (23), (24), (25), (28), (31), (32), (36)^. However, most did not specify the intensity of smoking or habitual use. While many acute CO poisoning cases involved two or more hours of waterpipe smoking, eight cases were reported after one hour or less ^(12), (14), (24), (31), (36)^. This suggests that factors such as depth of inhalation, smoking environment, and session duration play a significant role in the development of CO poisoning. In the case of polycythemia, habitual waterpipe smoking at least every other day appears to be a key factor.

Environmental conditions also contribute to CO poisoning risk. Poor ventilation can lead to CO poisoning ^(35)^ and increase the risk for both individuals who smoke actively and those passively exposed to waterpipe smoke ^(25), (28), (37)^. CO poisoning has also been reported in rooms where waterpipe smoking had previously taken place ^(31)^. Proper ventilation is essential, and waterpipe smoking should be conducted in outdoor or well-ventilated establishments that provide at least 130 m^3^ of fresh air per hour per waterpipe ^(47)^. However, outdoor smoking does not entirely eliminate CO poisoning risk, as several CO poisoning cases have still been reported under these conditions ^(12), (15), (16), (27), (36)^.

This study had several strengths and some limitations. One strength is that the systematic review of case reports helps identify rare cases and conditions and provides valuable insights into preventive measures for serious incidents. Additionally, the inclusion of case reports in foreign languages helped reduce the risk of overlooking important cases. However, this study had three major limitations. First, the quality of case reports was inconsistent. The descriptions in case reports may depend on the trade-off between the importance of information and word limits. In some cases, a lack of description does not necessarily indicate the absence of findings. Systematic surveillance using a structured questionnaire or checklist is required to assess the associations. Second, a systematic review of case reports does not represent prevalent cases, making the generalizability of these results difficult. Third, the AI-assisted systematic review is still a premature technique. Although the performance of GPT-4 for article selection and data extraction is reported to be high ^(48), (49)^, the accuracy is dependent on the prompt quality ^(50)^. Further methodological development is necessary to enhance the reproducibility and stability of AI assistance.

In conclusion, this study indicates that both active and passive waterpipe smoking can cause acute CO poisoning depending on the intensity, duration, and environment. Even with careful attention to the environment, it is impossible to completely avoid acute CO poisoning from waterpipe smoking. As symptoms may occur during or shortly after smoking but can also be delayed, waterpipe service workers need to pay attention to their customers’ conditions after they leave. The recommended level for indoor air CO was set at an 8-hour average of less than 50 ppm and less than 100 ppm at any time ^(51), (52)^, or more strictly ^(53)^. It is necessary to implement environmental regulations, such as setting a minimum ventilation air volume and requiring the installation of CO detectors, for closed establishments, such as waterpipe cafés and lounges, to prevent mass CO poisoning and protect workers from acute and chronic CO poisoning. Furthermore, when diagnosing CO poisoning, physicians need to consider waterpipe smoking as a possible cause. Though case reports provide insights, their findings are not necessarily generalizable. Further research is needed to determine the behavioral and environmental factors associated with waterpipe smoking.

## Article Information

### Acknowledgments

Use of generative artificial intelligence

We used the generative AI (ChatGPT Pro) primarily for the selection process, data extraction, and English editing. The author carefully verified all processes performed by the generative AI. The author analyzed the data and prepared all of the text, tables, and figures by himself.

### Author Contributions

Isao Muraki conceived and designed the study, collected, analyzed, and interpreted the data, and drafted the manuscript.

### Conflicts of Interest

None

### Ethical Review

As this study was a secondary data analysis of published articles, ethics committee review was not required.

### Data Availability Statement

Data is available (via email) from the corresponding author upon reasonable request.

## Supplement

Supplementary Material
