# Supplementary material for: Behavioral and Environmental Factors of Carbon Monoxide Poisoning and Polycythemia due to Waterpipe Smoking: An Artificial Intelligence-Assisted Systematic Review of Case Reports
Source: JMA J. 2026 Feb 20;9(2):447–56. doi: 10.31662/jmaj.2025-0208 (PMC13058741; doi:10.31662/jmaj.2025-0208)
Supplement: Supplementary Material [file 2433-3298-9-2_0447-s001.pdf]

Supplemental material 1

Selection process using ChatGPT

Prompt for selection process using ChatGPT o3-mini model

We are writing a systematic review of case report on waterpipe use and carbon monoxide poisoning/polycythemia. Select candidate articles from the list attached according to the following criteria and return the article number.

1. Relevance to Waterpipe (Shisha, Hookah, Narghile):
Does the title or abstract explicitly mention waterpipe or its synonyms (hookah, shisha, narghile)?
Yes / No

2. Clear Mention of Carbon Monoxide (CO) Poisoning or Related Outcomes:
Explicit mention of carbon monoxide poisoning or polycythemia.
Elevated carboxyhemoglobin (COHb) levels.
Symptoms consistent with acute carbon monoxide exposure (e.g., dizziness, nausea, headache, syncope).
Yes / No

3. Study Type Suitability:
Case reports, or case series (exclude editorials, reviews without original data, or general commentaries not containing case data).
Yes / No

Inclusion Decision:
Include: If Yes to all criteria (#1-3).
Exclude: If any criteria are marked No.

Supplemental material 2

Data extraction using ChatGPT

Prompt for data extraction using ChatGPT o3-mini-high model

Please extract the case-report information from the PDF text according to the following instructions and format:

Target

Include all case reports described in the PDF.

If multiple cases are presented, separate them into different rows (records) in the final table.

Data to Extract

Please create a table with the following columns. Where the information is not stated in the PDF, write “Not stated.”

File name

Authors (for example: “First author et al.”)

Publication (e.g., “Journal. Year; Volume: First page–Last page”)

Publication Year

Language

Country

Patient ID (if not explicitly provided, use something like “Case 1,” “Case 2,” etc.)

Age

Sex

Case types (e.g., Acute CO poisoning, Polycythaemia)

Shisha use

Active user or not

Co-user number

Place

Room size

Time using shisha

Using length (hrs)

Nicotine-free or not

Shisha café/bar worker or not

Alcohol use

Habitual shisha use frequency

Symptoms (list them, e.g., Headache, Dizziness, Vomiting, etc.)

Clinical information

Ambulance transport (yes/no)

GCS, JCS
Neurological exam
Physical exam
Brain CT, Brain MRI
Vital signs (BT, SBP, DBP, PR, RR, SpO2, BS, ECG, Hb, etc.)
Measured time after smoking
SpCO at triage or initial
SpCO after ABG
COHb at triage or initial
COHb after ABG
pH, pCO2, pO2, HCO3
Output Format

Provide the data in a structured table (in Markdown or CSV).
The first row should contain the column headers (the items listed above), and subsequent rows should contain the data for each case, one row per case.
If any piece of information is missing, please fill the cell with "Not stated."
Important Notes

If multiple cases appear, please ensure that all are included.
Use consistent wording and avoid potentially misleading abbreviations or footnotes.
For any information that the PDF omits or is unclear about, state it as "Not stated" or "Not clearly stated."
If you discover additional information (not in the predefined list) that may be relevant, you can add an extra "Additional Information" column.

Supplemental Table 1. List of included cases with acute carbon monoxide poisoning and polycythemia

				Demographics		Waterpipe Use				
ID	Authors	Year	Country	Age	Sex	Habitual use	Use before onset	Duration	Location	Co-users
Acute CO poisoning due to active smoking cases (single case)										
1	Lim et al[9]	2009	Singapore	19	M			4:00	Restaurant	Friends
2	Cavus et al[10]	2010	Turkey	25	M					
3	Arziman et al[14]	2011	Turkey	24	M					
4	Arziman et al[14]	2011	Turkey	27	F			3:00		Friends
5	Arziman et al[14]	2011	Turkey	17	F			0:30	Waterpipe café	
6	Höjer et al[12]	2011	Sweden	15	F		3 consecutive days	0:15		
7	Höjer et al[12]	2011	Sweden	28	M	Habitual			Balcony	
8	Höjer et al[12]	2011	Sweden	16	M			0:45	Outside	
9	Türkmen et al[13]	2011	Turkey	21	M			3:00	Closed room	
10	Türkmen et al[13]	2011	Turkey	20	F					
11	Uyanık et al[11]	2011	Turkey	25	M			2:00	Waterpipe café	
12	Ashurst et al[16]	2012	US	21	M				Outdoor	
13	La Fauci et al[15]	2012	Italy	16	F			3:00	Beach	Friends
14	Karaca et al[18]	2013	Turkey	20	F					
15	Ozkan et al[17]	2013	Turkey	19	M			4:00-5:00	Waterpipe café	Friends
16	von Rappard et al[19]	2014	Switzerland	16	M					Friends
17	von Rappard et al[19]	2014	Switzerland	21	M			Several hours		Friends
18	Stangl and Voigt[21]	2015	Denmark						Apartment	
19	Wang et al[20]	2015	Australia	20	F	Almost daily		1:00		
20	Ateş et al[22]	2016	Turkey	27	M			3:00-4:00	Home	
21	Paulsen et al[24]	2016	Denmark	18	M			Short		

								time		
22	Veen[23]	2016	Netherlands	15	M			1:30		
23	Veen[23]	2016	Netherlands	16	F			1:00		
24	Veen[23]	2016	Netherlands	28	M	3-4 times/wk		Several hours	Waterpipe bar	
25	Kocak et al[25]	2017	Turkey	20	M	2 times/mon	Smoking to prepare customer's waterpipe	2:00	Waterpipe café (worker)	
26	Kocak et al[25]	2017	Turkey	29	M	Occasional		3:00		
27	Kocak et al[25]	2017	Turkey	28	M			6:00		
28	de Suremain et al[29]	2019	France	13	M	Occasional			Basement	
29	Knörr et al[26]	2019	Germany	16	M				Apartment	Friends
30	Maalem et al[27]	2019	Saudi Arabia	43	M	Occasional			Outdoor	
31	Verweij et al[28]	2019	Netherlands	18	M			1:00		
32	Verweij et al[28]	2019	Netherlands	17	M					
33	Yamamoto et al[5]	2019	Japan	20	M			1:00	Restaurant	Friends
34	Cazes et al[31]	2021	France	32	M	Occasional		1:00+		
35	Cazes et al[31]	2021	France	23	F		Daily for a week		Apartment	
36	Supervía et al[30]	2021	Spain	17	F					
37	Supervía et al[30]	2021	Spain	23	M					
38	Supervía et al[30]	2021	Spain	36	F					
39	Gülçiçek et al[34]	2022	Netherlands	26	M				Waterpipe lounge (worker)	
40	Sunagawa et al[32]	2022	Japan	25	F		First time	3:00	Waterpipe bar	
41	Mohsen et al[33]	2023	Germany	21	M	3 times/wk			Home	
Acute CO poisoning due to active smoking cases (multiple cases)										
42	Arziman et al[14]	2011	Turkey	21	F			3:00-4:00	Waterpipe café	Cases 42-43

43	Arziman et al[14]	2011	Turkey		M			3:00-4:00	Waterpipe café	Cases 42-43
44	Clarke et al[35]	2012	UK	30	M				Basement party	Cases 44-55
45	Clarke et al[35]	2012	UK	34	M				Basement party	Cases 44-55
46	Clarke et al[35]	2012	UK	38	M				Basement party	Cases 44-55
47	Clarke et al[35]	2012	UK	26	M				Basement party	Cases 44-55
48	Clarke et al[35]	2012	UK	21	F				Basement party	Cases 44-55
49	Clarke et al[35]	2012	UK	21	F				Basement party	Cases 44-55
50	Clarke et al[35]	2012	UK	31	M				Basement party	Cases 44-55
51	Clarke et al[35]	2012	UK	19	F				Basement party	Cases 44-55
52	Clarke et al[35]	2012	UK	22	F				Basement party	Cases 44-55
53	Clarke et al[35]	2012	UK	17	F				Basement party	Cases 44-55
54	Clarke et al[35]	2012	UK	18	M				Basement party	Cases 44-55
55	Clarke et al[35]	2012	UK	22	F				Basement party	Cases 44-55
56	Bens et al[36]	2013	Netherlands	22	F			0:30	Festival	Cases 56-57
57	Bens et al[36]	2013	Netherlands	18	F			0:30	Festival	Cases 56-57
58	von Rappard et al[19]	2014	Switzerland	18	F			1:00	Waterpipe lounge	Cases 58-59
59	von Rappard et al[19]	2014	Switzerland	17	F			1:00	Waterpipe lounge	Cases 58-59
60	Supervía et al[30]	2021	Spain	42	M					Cases 60-62
61	Supervía et al[30]	2021	Spain	31	F					Cases 60-62
62	Supervía et al[30]	2021	Spain	36	F					Cases 60-62
63	Supervía et al[30]	2021	Spain	29	M					Cases 63-64
64	Supervía et al[30]	2021	Spain	28	M					Cases 63-64
Acute CO poisoning due to passive smoking cases										
65	Misek and Patte[37]	2014	US	24	M			No	Waterpipe bar (worker)	
66	Kocak et al[25]	2017	Turkey	18	M	No	3:00		Waterpipe café	Friends

67	Verweij et al[28]	2019	Netherlands	24	F		Staying in the room for several hours	Short time	Waterpipe lounge	
68	Cazes et al[31]	2021	France	43	F			No	Room where brother smoked	
Polycythemia cases										
69	Tadmor et al[38]	2011	Israel	Young	M	All days				
70	Bonadies et al[39]	2013	Switzerland	25	M	Daily		1:00-2:00		
71	Raaijmakers et al[40]	2020	Netherlands	22	M	Daily			Bedroom	
72	Moodley et al[41]	2021	South Africa	28	M	Daily		0:45		
73	Moodley et al[41]	2021	South Africa	29	M	2-3 times/d		1:00-1:30		
74	Moodley et al[41]	2021	South Africa	29	M	2-3 times/d		0:35		
75	Moodley et al[41]	2021	South Africa	47	M	2 times/d		1:05		
76	Moodley et al[41]	2021	South Africa	28	M	2-3 times/d		0:15		
77	Moodley et al[41]	2021	South Africa	31	M	3-5 times/d		0:30-0:45		
78	Moodley et al[41]	2021	South Africa	34	M	Daily		3:00		
79	Agbariah and Rovó[43]	2022	Switzerland	53	F	3 times/d				
80	Anaqrah and McCabe[42]	2022	US	51	M	3-4 times/d				
81	Sydorova et al[44]	2022	Ukraine	31	M	3-4 time/wk				

Abbreviations: CO, carbon monoxide

Gray cells indicate missing data.

Supplementary Table 1. List of included cases with acute carbon monoxide poisoning and polycythemia (continued)

ID	Symptoms<br>Onset	Syncope	Headache	Dizziness	Nausea or Vomiting	Impaired consciousness	Weakness or collapse	Dyspnea	Vertigo	Seizure	Chest pain	PE and DVT	Facial plethora	Others	None
Acute CO poisoning due to active smoking cases (single case)															
1				X										X	
2		X	X												
3	During use		X												
4	After use		X		X										
5	During use	X													
6	1 hour after use	X	X	X	X					X					
7	After use	X	X	X										X	
8	After use	X	X	X	X									X	
9	Shortly after leaving the room	X	X		x	X								X	
10	After use	X	X		X	X									
11			X		x				X						
12		X	X		x										
13	During use	X	X	X	X		X							X	
14	During use	X													
15	Shortly after use	X		X	X									X	
16	2 hours after use	X						X						X	
17	After use	X	X		X	X									
18		X													
19	After use		X	X	X	X								X	
20		X		X	X				X						
21		X													

22	After use		X		X							
23	After use			X	X			X				
24	During use	X		X	X							
25	After leaving the café	X	X	X	X							
26				X	X							X
27						X						X
28	1 night after use	X		X						X		
29		X	X		X							
30	During use		X		X	X	X					X
31	After use	X						X				X
32	After use	X		X	X		X					
33	After use	X										
34	During use	X										X
35			X									
36		X	X	X	X						X	
37		X		X								
38		X		X								
39	After work at shisha lounge	X						X			X	
40	After leaving the café	X					X					
41		X	X	X								
Acute CO poisoning due to active smoking cases (multiple cases)												
42					X	X			X			X
43												
44			X									

45			X								
46			X	X							
47			X								
48			X		X	X					
49				X							
50			X								
51											
52				X	X						
53			X	X							
54			X	X							
55				X							
56	After use	X	X	X						X	
57	After use		X								
58	After leaving the café	X	X							X	
59	No event										
60											X
61											X
62		X		X							
63				X	X						
64		X	X	X							
Acute CO poisoning due to passive smoking cases											
65	During use	X	X		X		X		X	X	
66	After leaving the café	X	X	X							
67	After use			X			X				
68	When case was in the room							X			

Polycythemia cases									
69								X	
70			X		X		X		
71									
72						X		X	
73				X					
74									
75									X
76						X			
77									
78		X		X					
79							X		
80									X
81									

Abbreviations: CO, carbon monoxide; DVT, deep vein thrombosis; PE, pulmonary embolism

‘X’ indicates applicable. Gray cells indicate missing data.

Supplementary Table 1. List of included cases with acute carbon monoxide poisoning and polycythemia (continued)

	Clinical information									Treatment		
ID	Mode of arrival	GCS	SBP/DBP (mmHg)	Pulse (bpm)	RR (bpm)	Neurological examination	SpCO (%)	COHb (%)	Hb (mg/dL)	Treatment	COHb after Tx (%)	Hb after Tx (mg/dL)
Acute CO poisoning due to active smoking cases (single case)												
1			108/61	99				27.8		NBO	1.1	
2						Normal		31.1		NBO	1.0	
3								21.0		NBO	1.7	
4			170/110	Tachycardia				20.2		NBO	3.6	
5						Normal		11.4		NBO	2.3	
6								21.0		NBO	1.0	
7								32.0		HBO		
8						Normal		23.7		NBO		
9		13	110/85	16	16	Normal		26.0		NBO	1.4	
10			120/80	103	15			27.5		NBO	1.5	
11						Normal		28.7	15.7	NBO	5.7	
12			138/80	97	22	Normal		15.3		NBO	1.3	
13	Ambulance	12	104/89	86				24.0		NBO		
14	Ambulance	15	70/40	96	26			31.1		NBO	1.0	
15	Ambulance		135/69		20			32.7		Monitoring	0.3	
16	Ambulance					Normal		20.1		NBO	4.8	
17	Ambulance	15				Normal		29.6		NBO	8.4	
18								18.0				
19	Ambulance	15	115/70	75		Normal		25.4		NBO		
20	Ambulance	15	125/70	79		Normal		32.6		NBO	1.0	
21								17.0		HBO	12.0	
22					22	Normal		25.0		NBO	7.4	

23	Ambulance		Normal	128	65			26.6		NBO	0.3	
24								39.2		NBO+HBO	3.7	
25	Ambulance	15						29.2		NBO	2.0	
26	On foot							34.1		NBO+HBO	3.0	
27	On foot	12						35.5		NBO+HBO	3.1	
28	On foot		116/60	94		Normal		23.1		NBO+HBO		
29		15	125/75	110	18			23.6	16.9	NBO	3.3	
30	Ambulance	14	134/67	71	22	Generalized fatigue and drowsiness, with mild left-sided weakness		38.8		NBO	2.8	
31	Ambulance					Normal		26.0		NBO	10.0	
32				115				19.0		NBO	7.0	
33	Ambulance	15	108/62	106	20	Normal		32.6		NBO	0.5	
34							27			NBO		
35							22			NBO		
36							40	10.8		HBO		
37								14.0		HBO		
38							38	17.4		HBO		
39			120/60	110	16			22.0		NBO	5.2	
40	Ambulance	6	123/87	74	18			30.7	13.8	NBO+HBO	0.4	
41	Ambulance	15	180/130	125	18			30.4		NBO+HBO		
Acute CO poisoning due to active smoking cases (multiple cases)												
42								21.3		NBO	3.8	
43								19.2		NBO	3.1	
44	On foot							15.0		NBO	2.8	

45	On foot							21.0		NBO	2.6	
46	On foot							18.0		NBO	1.8	
47	On foot							20.0		NBO	3.7	
48	Ambulance							18.0		NBO	<0.5	
49	Ambulance							7.3		NBO	2.0	
50	On foot							23.0		NBO	2.2	
51	Ambulance							2.0		NBO		
52	Ambulance							14.0		NBO	3.1	
53	Ambulance							12.0		NBO	4.9	
54	Ambulance							25.0		NBO	6.4	
55	Ambulance							15.0		NBO	2.1	
56								22.0		NBO	5.7	
57								19.5		NBO		
58	Ambulance	3				Normal		25.7		NBO	7	
59	Ambulance					Normal		16.7		NBO	5	
60							17	6.0		NBO		
61							10	2.9		NBO		
62							40	4.5		NBO		
63							31	25.9		HBO		
64							42	18.8		HBO		
Acute CO poisoning due to passive smoking cases												
65	Ambulance	15	134/71	105	18	Normal		33.8		NBO+HBO		
66	Ambulance	15						18.7		HBO	1.1	
67								26.0		NBO	9.0	
68							10			NBO		
Polycythemia cases												
69								27.2	22.7			
70			131/70	80				6.1	19.0	Waterpipe		17.7

									smoking reduction		
71							21.0	20.14	NBO	6.0	19.2
72								20.5			
73								18.9			
74								20.9			
75								21.5			
76							36.8	22.0			
77								19.4			
78								22.2			
79		140/85	100				22.7	18.6	Waterpipe smoking cessation		13.2
80		153/98	83	14	Normal		33.6	23.7	NBO	8.9	
81								16.6	Waterpipe smoking cessation		15.0

Abbreviations: bpm, beats or breaths per minutes; CO, carbon monoxide; COHb, carboxyhemoglobin; DBP, diastolic blood pressure; GCS, Glasgow Coma Scale; Hb, hemoglobin; HBO, hyperbaric oxygen therapy; NBO, normobaric oxygen therapy; RR, respiratory rate; SBP, systolic blood pressure; SpCO, peripheral carboxyhemoglobin saturation; Tx, treatment.

Gray cells indicate missing data.
